# The role of adverse childhood experiences and mental health care use in psychological dysfunction of male multi-problem young adults

**DOI:** 10.1007/s00787-018-1263-4

**Published:** 2018-12-14

**Authors:** Laura van Duin, Floor Bevaart, Josjan Zijlmans, Marie-Jolette A. Luijks, Theo A. H. Doreleijers, André I. Wierdsma, Albertine J. Oldehinkel, Reshmi Marhe, Arne Popma

**Affiliations:** 1 Department of Child and Adolescent Psychiatry, Amsterdam UMC, University of Amsterdam, Meibergdreef 5, 1105 AZ Amsterdam, The Netherlands; 2 Department of Psychiatry, Erasmus Medical Center, ‘s Gravendijkwal 230, 3015 CE Rotterdam, The Netherlands; 3 University Center Psychiatry, University of Groningen, University Medical Center Groningen, House Code CC 72, PO Box 30001, 9700 RB Groningen, The Netherlands

**Keywords:** Adverse childhood experiences, Mental health care use, Young adults, Multi-problem, Psychological dysfunction

## Abstract

Adverse childhood experiences (ACEs) are associated with severe problems later in life. This study examines how eleven types of ACEs and mental health care use history are related to current psychological dysfunction among multi-problem young adults. A sample of 643 multi-problem young adult men (age 18–27) gave informed consent for us to collect retrospective regional psychiatric case register data and filled out questionnaires. ACEs were highly prevalent (mean 3.6, SD 2.0). Logistic regression analysis showed that compared with participants who experienced other ACEs, participants who experienced psychological problems in their family and grew up in a single-parent family were more likely to have used mental health care, and physically abused participants were less likely to have used mental health care. Linear regression analyses showed a dose–response relationship between ACEs and internalizing and externalizing problems. Linear regression analyses on the single ACE items showed that emotional abuse and emotional neglect were positively related to internalizing problems. Emotional and physical abuse and police contact of family members were positively related to externalizing problems. While multi-problem young adults experienced many ACEs, only a few ACEs were related to mental health care use in childhood and adolescence. Long-term negative effects of ACEs on psychological functioning were demonstrated; specifically, emotional abuse and emotional neglect showed detrimental consequences. Since emotional abuse and emotional neglect are not easily identified and often chronic, child health professionals should be sensitive to such problems.

## Introduction

Adverse childhood experiences (ACEs), such as abuse, neglect and household dysfunction, are considered negative exposures that have a broad impact on child development [1, 2] and on the occurrence of mental health problems later in life [1, 3, 4]. According to the World Health Organization (WHO) World Mental Health Surveys, ACEs account for 29.8% of all psychiatric disorders [5]. Multi-problem young adults suffering from an accumulation of psychological problems/disorders and substance abuse and experiencing court involvement are likely to have experienced one or more ACEs during childhood [4, 6–12]. Studies that use stress process frameworks, such as the Stress Process Model [13, 14], as a theoretical basis have made a valuable contribution to deepening our understanding of the ‘chain of succeeding events’ that can often be observed within the life course of individuals from families in which ACEs frequently occur. Such frameworks incorporate the influence of stressors at the individual, family, and community levels with a focus on predicting mental health outcomes. These studies acknowledge that dimensions of mental health vary based on social determinants such as socioeconomic status (SES), household factors, migration status and social capital. An important task is to identify the conditions that help explain these variations, for example, by assessing childhood problems and subsequent access to specialist mental health care in specific populations of vulnerable (young) adults with mental health problems. Mental health care utilization as an outcome measure in earlier studies is rare and mainly based on self-reported recall [15, 16]. Therefore, the aim of this study was to retrospectively explore how abuse, neglect and household dysfunction were related to registered mental health care use history within a high-risk sample of multi-problem young adults from a disadvantaged socioeconomic background. Additionally, we investigated the association between ACEs and two measures of current psychological functioning within this group, and we examined how mental health care use contributed to this relationship. This paper contributes to the evolving understanding of ACEs, focusing on understanding their relation with mental health care use and their influence on young adult mental health problems.

 ACEs have been extensively investigated in The Adverse Childhood Experiences (ACE) Study [4] and are commonly defined by three categories: abuse, neglect and household dysfunction [4]. To date, most studies have investigated ACEs within general population samples [2, 5, 12, 17–22], among adult members of a health maintenance organization (HMO) [3, 8, 9, 23–26], and within high-risk groups [6, 10, 27–29], such as individuals within the juvenile justice system [29], individuals convicted for offenses [30], and children who grew up with incarcerated family members [31]. The current study focused on a very specific population of high-risk men (aged 18–27 years) who grew up in socially disadvantaged circumstances and, during their youth, developed severe and intertwined problems in several important life domains (‘multi-problem young adults’). A previous study performed on this sample of multi-problem young adults showed that 87% belonged to an ethnic minority group, 63% reported severe family problems during their youth, and 66% had experienced at least one Child Protection Services (CPS) contact during their youth, mainly for judicial reasons [32]. An ethnic minority background and juvenile delinquency are both strongly related to childhood poverty, deprivation and developmental problems [33, 34]. According to social stratification theory and social control theory, crime rates are higher in disadvantaged communities as a result of a lack of social integration due to income inequality, which can generate strain, frustration and a lack of informal social control [35]. A large body of recent literature shows a strong intertwined relationship between growing up socially disadvantaged and experiencing ACEs. A study of children with CPS involvement showed that poverty was strongly related to neglect. Physical neglect was predicted by parental substance use problems and mental health problems [36]. Therefore, problems referred to as household dysfunction, including parenting problems, were related to (types of) maltreatment. In a study of young adults, neglect and poverty in childhood were related to post-traumatic stress disorder and arrest in adulthood. Poverty was also related to depressive disorders in adulthood [37]. A retrospective study on detainees showed that the experience of multiple types of maltreatment was related to a low socioeconomic status in childhood, growing up in a single parent family, substance abuse of family members and a history of suicide attempts. Emotional and physical neglect were more prevalent among detainees with a convicted family member, whereas emotional abuse was related to psychiatric disorders [38]. Both separate and cumulative effects of ACEs on adult health problems have been observed [39]. Estimating the prevalence and the long-term impact of ACEs on mental health and care use for these high-risk young adults is important in terms of establishing their specific need for mental health care and identifying potential barriers to (specialist) mental health care use in vulnerable populations.

 Within the widely used main categories, eleven ACEs can be distinguished: emotional, physical, and sexual abuse; emotional and physical neglect; and household dysfunction, which can be subdivided into growing up in a single parent family, domestic violence, family members’ police contact, drug and alcohol abuse, and psychological problems of a family member [4, 40]. The three distinct clusters of ACEs are interrelated and strongly associated with socioeconomic background [36, 37, 41, 42], (mental) health, well-being, and overall self-sufficiency in young adulthood [4, 28, 43]. Young adulthood (18–27 years) is now widely acknowledged as a developmental stage that includes major psychological [44–46], social [44] and neurobiological [47] changes that are critical for a healthy transition to adulthood [48–50]. Young adults with severe childhood problems are at increased risk of unemployment, early parenthood, delinquent behaviour, and substance abuse [28, 38, 51–55]. Therefore, a high prevalence of ACEs can be expected within the retrospective reports of multi-problem young adults. Since ACEs are strongly associated with the development of mental health problems during childhood and adolescence [5], they may result in the use of professional services [56], including mental health care [39, 53, 57]. There is also a large body of evidence that these mental health services are not routinely accessible to low-income and marginalized populations [58–61]. Socioeconomic and stigma-related barriers impede parents from ethnic minority backgrounds from obtaining mental health services for themselves and their children [59]. A study within a large high-risk population of young children with mental health problems showed an underrepresentation of ethnic minority children in the Dutch mental health care system [62]. To date, a number of studies have indicated that ACEs are associated with higher levels of health care utilization in adulthood [39, 63], and some studies have investigated the relation between ACEs and mental health care use specifically. A study on female offenders showed that ACEs have a strong and cumulative effect on health outcomes and significantly increase mental health care use in adulthood [29]. Other studies have shown that emotional, physical, or sexually abused children more frequently use mental health care services than non-maltreated children [17, 64]. Likewise, children living in a single-parent family or with parents who have psychological problems have an increased likelihood of receiving mental health care compared to children experiencing less household dysfunction [22, 65]. However, the literature is not conclusive on the association between ACEs and mental health care use. Some studies have shown that adolescents and adults with ACEs are especially impeded to start mental health care use [66, 67], possibly due to a lack of awareness that their mental health problems are derived from ACEs [66]. Previous studies have indeed shown that a minority of children and adolescents with multiple ACEs receive mental health care [65–69], therefore, their needs and use do not appear to correspond. Furthermore, it is unknown how neglect and service use are related. Other factors, such as parent–child conflicts, juvenile delinquency, self-reported problems, family stress and parental problem perception, tend to be stronger predictors of the receipt of treatment for mental health problems than ACEs [65, 67, 68]. Within our sample of multi-problem young adults, we will explore the association between ACEs and mental health care use in childhood and adolescence. Using such a high-risk sample provides an important opportunity to investigate the unique contribution of the distinct types of ACEs to this association in more depth.

 Regarding the association between ACEs and later psychological functioning, most studies have focused on specific disorders in relation to ACEs without taking previous mental health care use into account. Prior studies on disorders found that adults with severe depression reported significantly more emotional abuse than adults with less severe depression [70], and emotional, physical and sexual abuse are considered risk factors in the development of later dysthymia [8, 27]. Household dysfunction, specifically parental substance abuse, psychological problems, police contact and growing up in a single-parent family, are associated with suicide attempts [25]. Emotional abuse is considered the strongest predictor of lifetime depressive disorders and suicide attempts [8, 25, 71]. Furthermore, physical and sexual abuse [19, 27], neglect [27], living in a single-parent family [2], and adult alcohol abuse [26] are related to antisocial personality disorder, substance abuse and delinquent behaviour. Hence, several ACEs within the maltreatment and household dysfunction categories have been associated with the development of both internalizing and externalizing problems [20, 25, 26, 72]. On the other hand, the association between neglect and later mental health problems is somewhat more ambivalent: one study showed a relation with internalizing and externalizing problems [27], whereas Keyes et al. [19] failed to reproduce this result, and Hunt et al. [2] concluded that only physical neglect and internalizing problems are related.

 So far, few studies have explored the relation between ACEs, internalizing problems and externalizing problems together. The studies that did combine these measures showed that physical abuse [2, 19] and police contact of family members [2] are specifically related to externalizing problems, whereas physical neglect [2] and emotional abuse [19] are related only to internalizing problems. Therefore, it is expected that distinct types of ACEs are related differently to internalizing and externalizing problems. Concerning our male population, it is shown that young adult men have health risk profiles that are distinct from those of young women [73]. These gender differences underscore the need to target (high-risk) young adult men to increase their knowledge about their mental health care needs. Improved knowledge on the relations between distinct types of ACEs and internalizing and externalizing problems and the contribution of mental health care use to these problems would afford insight into the development of psychological problems among multi-problem young adults and the tailoring of mental health care to meet their needs. To our knowledge, this study is the first study to relate ACEs and mental health care use with internalizing and externalizing problems in young adulthood.

 The present study aims to retrospectively describe the prevalence of distinct types of ACEs (i.e. abuse, neglect, and household dysfunction) in a group of multi-problem young adults and to examine whether these ACEs are differentially associated with mental health care use in childhood and adolescence. Subsequently, the association between distinct ACEs, previous mental health care use and current internalizing and externalizing problems are explored. We expect that mental health care use is positively associated with ACEs in general and living in a single-parent family and parental psychological problems in particular [22, 64, 65]. Furthermore, we expect current internalizing problems and externalizing problems to be related to both ACEs and mental health care use. A dose–response relationship between ACEs and internalizing and externalizing problems is expected, as well [8, 39]. More specifically, a positive association between internalizing problems and ACEs is expected for (emotional) abuse and household dysfunction in general, and a positive association between externalizing problems and ACEs is expected for (physical) abuse and household dysfunction in general [2, 19]. Moreover, residential mobility and ethnicity are two intertwined factors related to ACEs, mental health care use and internalizing and externalizing problems in young adulthood [74–76]. Residential mobility is positively associated with the number of ACEs [75, 77, 78] and with a reduced continuity of healthcare [79]. Furthermore, the mental health care use of ethnic minorities tends to be lower than that of ethnic majority groups [80]. Most multi-problem young adults belong to ethnic minority groups, and due to their various family and income problems, we expect their residential mobility to be relatively high [81]. Therefore, the influence of residential mobility and ethnicity is taken into account when exploring the associations between ACEs, mental health care use and current mental health problems.

## Methods

### Subjects and setting

In 2014–2016, a total of 647 multi-problem young adult men were recruited in Rotterdam, The Netherlands, as part of a larger study [54, 81]. Recruitment for this study took place at two sites. The first site was the multimodal day treatment programme New Opportunities (Dutch: *De Nieuwe Kans*; DNK), where 173^1^ participants were recruited. DNK offers a multimodal intervention for multi-problem young adult males, therefore, we included only men. Participants signed up for DNK themselves or were referred to DNK directly by youth care, probation services, mental health services, or social organizations. The second site was the municipal agency in Rotterdam (Dutch: *Jongerenloket*), where young adults between the ages of 18 and 27 can apply for social welfare. During the intake, the self-sufficiency of all young adults is scored by a youth coach on eleven life domains with the Self-Sufficiency Matrix—Dutch version (SSM-D) [82–85]. The SSM-D has scores of 1 (in crisis), 2 (vulnerable), 3 (stable), 4 (safe) and 5 (thriving). After the intake, a statutory effort period of 4–6 weeks is followed. Within this period, the young adult is obliged to try to find education or work. He is referred to an intervention such as DNK when he does not meet these aims and meets the conditions for acquiring social welfare. Participants were eligible when they were male, were between 18 and 27 years old (mean age 22.1; SD = 2.4), and adhered to the following definition of multi-problems: (a) a score of 1 or 2 on the domains Income and Daytime Activities; (b) a maximum score of 3 on at least one of the following domains: Addiction, Mental Health, Social Network, and Justice; and (c) a minimum score of 3 on the domain Physical Health [81]. This definition was based on the self-sufficiency scores in the prior year of all young adult males who were referred to DNK. At the municipal agency, 474^2^ participants were recruited. Of the total study sample (*N* = 647), *N* = 643 (99.4%) gave informed consent to the record and register research. The study was approved by the Medical Ethics Review Committee of VU University Medical Center.^3^ Participants gave informed consent for their voluntary participation after a member of the research team provided oral and written information. After informed consent was obtained, trained researchers administered the questionnaires by means of an interview.

### Measurements

#### Demographic characteristics

Ethnicity was based on the country of birth of the participant and his parents. Rotterdam has a large proportion of non-Dutch inhabitants. The largest groups in The Netherlands originally migrated from Morocco and Turkey as labour migrants in the 1960s and early 1970s. During the process of decolonization after 1975, Surinamese and Antillean migrants came from South America and the Caribbean to The Netherlands. Ethnicity was recoded into eight categories: Dutch, Moroccan, Antillean, Surinamese, Cape Verdean, Turkish, other Western, and other non-Western. If the country of birth of at least one of the parents or the participant was outside The Netherlands, he was classified as non-Dutch, conforming to the Dutch CBS definition [86]. A dichotomized variable (Dutch Yes/No) was used in the regression analyses. Residential mobility (number of movements) was assessed with the residential mobility calendar [87].

#### Adverse childhood experiences

Exposure to ACEs was operationalized using eleven types of experiences falling within three categories: abuse, neglect, and household dysfunction. Additionally, we summed a total ACE score that referred to the participants’ first eighteen years of life and matched the categorization used in the ACE study as closely as possible [24], with a few exceptions. The exceptions were as follows: we used police contact of family members instead of incarceration (as in the ACE study), and we measured alcohol abuse and drug abuse separately instead of using one combined measure for substance abuse (as in the ACE study).

#### Maltreatment

The 24-item Dutch Childhood Trauma Questionnaire-Short Form (CTQ-SF) was used to assess the frequency of five ACEs [88]: physical abuse, emotional abuse, sexual abuse, physical neglect, and emotional neglect. The item response categories were Likert-type and scored from one to five (never true, rarely true, sometimes true, often true, very often true). Abuse was classified as none, low, moderate, or severe [88]. The data from the CTQ-SF were severely skewed, so we dichotomized the variables using cut-off scores in which low, moderate, and severe are considered abuse, as applied in Bernstein et al. [89] and in Walker et al. [90]. For physical abuse and physical neglect, a score above 7 was categorized as low to severe. For sexual abuse, emotional neglect, and emotional abuse, scores above 5, 9, and 8, respectively, were categorized as low to severe [89, 90].

#### Household dysfunction

Household dysfunction consists of six ACEs: alcohol abuse in the family, drug abuse in the family, police contact in the family, psychological problems in the family, domestic violence, and growing up in a single-parent family. Alcohol abuse problems in the family were assessed with the item ‘Did you suffer from alcohol abuse problems that existed in the family you grew up with? (Yes/No)’. Drug abuse problems in the family were assessed with the item ‘Did you suffer from drug abuse problems that existed in the family you grew up with? (Yes/No)’. Police contact of family members in youth was assessed with the item ‘Did family members you grew up with have police contact? (Yes/No)’. Psychological problems of family members were assessed with the item ‘Did you suffer from psychological problems that existed in the family you grew up with? (Yes/No)’. Witnessing domestic violence was assessed with the item ‘Did you suffer from domestic violence in the family you grew up with? (Yes/No)’. Growing up in a single-parent family was assessed with the residential mobility calendar [87] using the question ‘Who was residing in the household when you were five years old?’ By writing down each person (father, mother, brother, sister, etc.), the household composition was determined, and a new variable was computed (single parent: Yes/No).

#### Use of mental health care

Data on the use of mental health care in childhood and adolescence were extracted from the Psychiatric Case Register (PCR) Rotterdam Region. A psychiatric case register is a “patient-centered longitudinal record of contacts with a defined set of psychiatric services, originating from a defined population” [91]. The PCR Rotterdam Region contains information on all mental health care services in the area until 2010; these services include the Regional Institutes for Outpatient Mental Health Care, other outpatient services and clinics for psychiatric care, crisis intervention services, sheltered homes, day centres, and (general) psychiatric hospitals. The register data were linked to the multi-problem young adults with the probabilistic linkage method using the first two letters of the last name, date of birth, gender, nationality and postal code as identifiers [92]. The case register provided information on whether the participants received mental health care in youth and adolescence. We also collected more detailed information on mental health care use, such as the age of the first contact and the total number of contacts. Mental health care use was used as a dichotomous outcome measure (Yes/No).

#### Current psychological functioning

To assess current psychological functioning, the Dutch version of the Adult Self-Report (ASR) [93] was used. The ASR (part VIII) measures internalizing and externalizing problems during the previous 6 months with 123 items. We used the internalizing problem score and the externalizing problem score as outcome measures.

### Analysis

Multiple statistical analyses were performed using Statistical Package for the Social Sciences (SPSS) version 21 [94]. First, logistic hierarchical regression analysis was executed to investigate associations between eleven ACEs and mental health care use in childhood and adolescence, controlling for ethnicity, the number of movements, and age (mean centred). Second, logistic hierarchical regression analysis was executed to investigate associations between the total number of ACEs and mental health care use in childhood and adolescence, controlling for ethnicity, the number of movements, and age (mean centred). The outcome measure was mental health care use (Yes/No). As model statistics, the area under the curve (AUC) value and Hosmer–Lemeshow’s measure were computed. An AUC value of 1 signifies perfect classification, and a value of 0.50 indicates classification accuracy equal to chance. The variables were entered into the regression in two steps: (1) the control variables (ethnicity, number of movements and age) and (2) the ACEs (emotional, physical, and sexual abuse; emotional and physical neglect; alcohol abuse in the family; drug abuse in the family; police contact in the family; psychological problems in the family; domestic violence; and growing up in a single-parent family). Third, two linear hierarchical regression analyses were performed to explore the association between the eleven ACEs and (1) internalizing and (2) externalizing problems in young adulthood as outcome measures, controlling for ethnicity, number of movements, and age (mean centred). In the first step, the control variables were added. In the second step, all ACEs were added. In the third step, mental health care use (Yes/No) was entered to explore whether prior mental health care, in addition to ACEs, was related to current internalizing and externalizing problems. Fourth, two similar linear hierarchical regression analyses were performed with the total number of ACEs as the independent variable.

## Results

Table 1 shows the prevalence of self-reported demographic characteristics and ACEs, registered mental health care use in childhood and adolescence, and current internalizing and externalizing problems of multi-problem young adults. The majority of the participants were non-Dutch (88.1%): 19.8% were Moroccan, 17.8% were Antillean, 17.8% were Surinamese, 8.2% were Cape Verdean, 6.6% were Turkish, 4.5% had another Western background, 13.4% had another non-Western background, and 11.9% were Dutch. ACEs were highly prevalent: 99.8% had experienced at least one ACE. A mean of 3.6 ACEs was reported, and emotional neglect was the most frequently reported ACE (69.1%). Of the total sample (*N* = 643), one-third (*N* = 197) had used mental health care in childhood and adolescence. In addition to the mean scores on internalizing and externalizing problems shown in Table 1, we calculated the prevalence of current (borderline) clinical dysfunction based on ASR percentile scores. The results showed that 42.2% of the sample reported (borderline) clinical internalizing problems, and 29.9% reported serious externalizing problems [95].

**Table 1:** Percentage or mean score of demographic characteristics, ACEs and outcome measures (*N* = 643)

	Percentage yes or Mean Score	SD
Demographic characteristic		
Ethnicity (%)		
Dutch	11.9	
Non-Dutch	88.1	
Mean age	22.1	2.4
Residential mobility—mean number of movements^a^	4.4	3.7
Adverse childhood events (ACEs)		
Maltreatment		
Emotional abuse (%)^b^	33.1	
Physical abuse (%)^c^	34.3	
Sexual abuse (%)^c^	10.1	
Emotional neglect (%)^c^	69.1	
Physical neglect (%)^b^	39.2	
Household dysfunction		
Single-parent family (%)^a^	40.2	
Family problems—alcohol abuse (%)	11.0	
Family problems—drug abuse (%)	9.2	
Family problems—police contact (%)	16.2	
Family problems—psychological problems (%)	9.6	
Family problems—domestic violence (%)	13.7	
Mean total ACEs	3.6	2.0
Registered mental health care use		
Mental health care use (%)	30.6	
Mean age first contact^d^	12.6	3.9
Mean number of contacts^e^	23.5	43.2
Psychological functioning previous 6 months		
Mean score internalizing problems	69.2	26.6
Mean score externalizing problems	65.1	26.1

^a^*N* = 642; ^b^*N* = 640; ^c^*N* = 641; ^d^*N* = 183; ^e^*N* = 195

### ACEs and mental health care use in childhood and adolescence

The first logistical hierarchical regression analysis was conducted to examine the association between different types of ACEs and mental health care use, controlling for ethnicity, age, and number of movements (see Table 2). The model statistics showed the AUC (.629) and the Hosmer–Lemeshow test (4.43) (see note Table 2). Of the eleven types of ACEs that were included in the analyses, only physical abuse, psychological family problems and growing up in a single-parent family were significantly associated with having used mental health care during childhood and adolescence. These results indicate the following: (1) participants who were physically maltreated were less likely to have used mental health care than those who had experienced other ACEs; (2) participants who reported psychological family problems were more likely to have used mental health care; and (3) multi-problem young adults who grew up in a single-parent family were more likely to have used mental health care than those who did not grow up in a single-parent family. The second logistic hierarchical regression showed that the total number of ACEs was not associated with mental health care use.

**Table 2:** Logistic regression analysis ACEs and mental health care use (*N* = 640)

Predictors	*B* (SE)	95% CI for odds ratio		
		Lower	Odds ratio	Upper
Step 1				
Constant	− 0.567 (0.256)		0.567	
Ethnicity	− 0.238 (0.257)	0.476	0.788	1.304
Age	0.046 (0.036)	0.976	1.048	1.125
Number of movements	− 0.013 (0.024)	0.942	0.987	1.035
Step 2				
Constant	− 0.560 (0.302)		0.571	
Ethnicity	− 0.216 (0.277)	0.468	0.806	1.387
Age	0.053 (0.037)	0.980	1.054	1.134
Number of movements	− 0.008 (0.026)	0.942	0.992	1.045
ACE single items				
Emotional abuse	− 0.194 (0.243)	0.511	0.824	1.327
Physical abuse	− 0.654 (0.232)	0.330	0.520**	0.820
Sexual abuse	− 0.289 (0.328)	0.394	0.749	1.425
Emotional neglect	− 0.072 (0.207)	0.619	0.930	1.397
Physical neglect	0.072 (0.203)	0.722	1.075	1.599
Family problems—alcohol abuse	0.109 (0.349)	0.562	1.115	2.213
Family problems—drug abuse	− 0.488 (0.401)	0.280	0.614	1.346
Family problems—police contact	0.008 (0.286)	0.576	1.008	1.765
Family problems—psychological problems	0.881 (0.340)	1.240	2.413*	4.696
Family problems—domestic violence	0.328 (0.313)	0.751	1.388	2.566
Single-parent family	0.388 (0.184)	1.028	1.473*	2.111
Total number of ACEs	− 0.031 (0.298)	0.886	0.970	1.061

Statistics for model including ACE single items. Step 1: *R*^2^ = .006 (Nagelkerke). Model *χ*^2^ (3) = 2.655, n.s. Step 2: *R*^2^ = .06. Model *χ*^2^ (14) = 27.51, *p* < .05. AUC = .629 (SE 0.024), *p* < .001; Hosmer–Lemeshow test: 4.43 (*df* = 8), *p* = .816

Statistics for model including total ACEs. Step 1: *R*^2^ = .007 (Nagelkerke). Model *χ*^2^ (3) = 3.064, n.s. Step 2: *R*^2^ = .008. Model *χ*^2^ (4) = 3.52, n.s. AUC = .629 (SE 0.024), *p* < .001; Hosmer–Lemeshow test: 6.99 (*df* = 8), *p* = .537

*p < .05; **p < .01; ***p < .001

### ACEs and mental health care use in relation to current internalizing problems

Linear hierarchical regression analysis was executed to analyse the association between ACEs and current internalizing problems, controlling for ethnicity, number of movements and age (Table 3). The control variables (step 1) accounted for 4% of the explained variance in current internalizing problems (*F *= 9.69; *R*^2^= .04; *p *< .001). The addition of ACEs (step 2) added a significant 10% of explained variance to the model (*F *= 7.15; *R*^*2*^= .14; *p *< .001). Adding mental health care use (step 3) did not significantly change the fit of the model. Regarding the individual associations between the predictors and internalizing problems, emotional abuse and emotional neglect were positively associated with current internalizing problems. This indicates that emotional abuse and emotional neglect during childhood are associated with more internalizing problems in young adulthood. The analyses with the total number of ACEs as predictor variables showed a positive significant association in steps 2 and 3, indicating a dose–response relationship between ACEs and internalizing problems in young adulthood.

**Table 3:** Linear regression analysis ACEs, mental health care use and current internalizing problems (N = 639)

Predictors	*B*	SE *B*
Step 1		
Constant	71.975	3.161
Ethnicity	− 7.701*	3.162
Number of movements	0.944**	0.283
Age	1.259**	0.432
Step 2		
Constant	61.479	3.480
Ethnicity	− 4.184	3.185
Number of movements	0.389	0.292
Age	1.251**	0.415
ACE single items		
Emotional abuse	9.478***	2.639
Physical abuse	3.111	2.485
Sexual abuse	3.500	3.384
Emotional neglect	7.068**	2.364
Physical neglect	1.616	2.257
Family problems—alcohol abuse	3.680	3.842
Family problems—drug abuse	− 1.913	4.301
Family problems—police contact	2.730	3.157
Family problems—psychological problems	0.298	3.889
Family problems—domestic violence	0.266	3.421
Single-parent family	− 2.413	2.066
Total number of ACEs	3.581***	0.527
Step 3		
Constant	62.500	3.567
Ethnicity	− 4.310	3.185
Number of movements	0.384	0.292
Age	1.282**	0.416
ACEs single items		
Emotional abuse	9.372***	2.639
Physical abuse	2.752	2.500
Sexual abuse	3.353	3.384
Emotional neglect	7.025**	2.363
Physical neglect	1.660	2.256
Family problems—alcohol abuse	3.744	3.841
Family problems—drug abuse	− 2.178	4.303
Family problems—police contact	2.744	3.155
Family problems—psychological problems	0.814	3.907
Family problems—domestic violence	0.441	3.422
Single-parent family	− 2.189	2.072
Mental health care use	− 2.808	2.186
Total number of ACEs	3.556***	0.526

Statistics for model including ACEs single items. *R*^2^ = .04*** for Step 1; *R*^2^ = .14***, Δ*R*^2^ = .10*** for Step 2 (*df* = 14); *R*^2^ = .14***, Δ*R*^2^ = n.s. for Step 3

Statistics for model including total ACEs. *R*^2^ = .04*** for Step 1; *R*^2^ = .11***, Δ*R*^2^ = .07*** for Step 2 (*df* = 4); *R*^2^ = .11***, Δ*R*^2^ = n.s. for Step 3

*p < .05; **p < .01; ***p < .001

### ACEs and mental health care use in relation to current externalizing problems

Linear hierarchical regression analysis was executed to analyse the association between ACEs and current externalizing problems, controlling for ethnicity, number of movements and age (Table 4). The control variables (step 1) accounted for 5% of the explained variance in current externalizing problems (*F *= 12.09; *R*^2^= .05; *p *< .001). The addition of ACEs (step 2) added a significant 14% of explained variance to the model (*F *= 10.58; *R*^2^= .19; *p *< .001). Adding mental health care use (step 3) did not significantly change the fit of the model. Regarding the individual associations between the predictors and externalizing problems, emotional abuse, physical abuse and police contact of family members was positively associated with current externalizing problems. This finding indicates that emotional abuse, physical abuse and police contact of family members during childhood are associated with more externalizing problems in young adulthood. The analyses with the total number of ACEs showed a positive significant association in steps 2 and 3, which demonstrates a dose–response relationship between ACEs and externalizing problems in young adulthood.

**Table 4:** Linear regression analysis ACEs, mental health care use and current externalizing problems (*N* = 639)

Predictors	*B*	SE *B*
Step 1		
Constant	71.304	3.082
Ethnicity	− 12.755***	3.083
Number of movements	1.152***	0.276
Age	0.456	0.421
Step 2		
Constant	61.071	3.303
Ethnicity	− 8.205**	3.023
Number of movements	0.491	0.277
Age	0.463	0.394
ACE single items		
Emotional abuse	11.075***	2.505
Physical abuse	6.721**	2.359
Sexual abuse	3.578	3.212
Emotional neglect	2.943	2.244
Physical neglect	− 1.639	2.142
Family problems—alcohol abuse	4.523	3.647
Family problems—drug abuse	3.998	4.082
Family problems—police contact	7.575*	2.996
Family problems—psychological problems	− 1.120	3.691
Family problems—domestic violence	1.193	3.247
Single-parent family	− 1.832	1.961
Total number of ACEs	4.118***	0.505
Step 3		
Constant	62.141	3.385
Ethnicity	− 8.337**	3.022
Number of movements	0.486	0.277
Age	0.495	0.395
ACE single items		
Emotional abuse	10.965***	2.504
Physical abuse	6.345**	2.372
Sexual abuse	3.424	3.211
Emotional neglect	2.897	2.242
Physical neglect	− 1.593	2.140
Family problems—alcohol abuse	4.590	3.644
Family problems—drug abuse	3.722	4.084
Family problems—police contact	7.590*	2.994
Family problems—psychological problems	− 0.579	3.708
Family problems—domestic violence	1.376	3.247
Single-parent family	− 1.597	1.966
Mental health care use	− 2.941	2.074
Total number of ACEs	4.089***	0.504

Statistics for model including ACEs single items. *R*^2^ = .05*** for Step 1; *R*^2^ = .19***, Δ*R*^2^ = .14*** for Step 2 (*df* = 14); *R*^2^ = .19***, Δ*R*^2^ = n.s. for Step 3

Statistics for model including total ACEs. *R*^2^ = .05*** for Step 1; *R*^2^ = .14***, Δ*R*^2^ = .09*** for Step 2 (*df* = 4); *R*^2^ = .15***, Δ*R*^2^ = .01* for Step 3

*p < .05; **p < .01; ***p < .001

## Discussion

The purpose of this study was threefold. The first aim was to establish the prevalence of ACEs in multi-problem young adult men. As expected in a high-risk population [43] with a disadvantaged socioeconomic and non-Dutch background, ACEs were highly prevalent in our sample: 99.8% reported at least one ACE, and they experienced 3.6 ACEs on average. Emotional neglect was the most common ACE in the sample: 69.1% reported emotional neglect (versus 12.4% of adult men in the general population [4]). Furthermore, emotional and physical abuse, physical neglect, police contact of a family member, domestic violence and growing up in a single-parent family were all more prevalent in multi-problem young adults than in a general population of adult men in the ACE study [4]. The second aim was to examine the association between distinct types of ACEs and mental health care use during childhood and adolescence in multi-problem young adults. In this study, 30.6% of the sample had received mental health care in childhood and adolescence. Our expectation (based on previous research) that ACEs are likely to affect the receipt of mental health care was, therefore, partly confirmed [65]. However, these results must be interpreted with caution since the total ACE exposure analysis was not significant, and the model-AUC of the association between ACEs and mental health care use was only .629. Since we used a dichotomous measure of ACEs, this AUC value might indicate that when clinical details on ACEs such as age of exposure, frequency, severity and duration are available, the clinical relevance might increase. The results further showed that only a few specific ACEs were related to mental health care use. A possible explanation for the weak associations between ACEs and the use of mental health care found in our study is that other social determinants led to the limited use of mental health care; such determinants are poverty, household stressors and low social capital. It has been suggested that these factors increase the risk of toxic stress and consequently decrease the chance of using specialist services [96]. Neglect and abuse were not associated with mental health care use in our sample, with physical abuse as the only exception. However, physically abused participants were less likely to have used mental health care. An explanation for the findings may be that children are often abused by a parent, which may result in less problem recognition due to physical abuse and thereafter less help-seeking by parents [97]. In addition, stigma perceived by parents about mental health problems and care services [59, 61] and concerns about the condemnation of their parental skills as a consequence of CPS contact may result in less help-seeking. Furthermore, two ACEs within the category of household dysfunction were related to mental health care use: psychological problems of a family member and growing up in a single-parent family. This finding corresponds to a study that found that 6 to 9-year-old children growing up in a single-parent family or stepfamily household were more likely to use mental health care [65]. Most likely, a single parent is more often inclined to seek mental health service for a child due to a lack of support from the other parent [68]. Lastly, in line with previous studies, psychological problems of a family member increased the likelihood of their children using mental health care [22]. A first explanation may be that children of parents with psychological problems have an increased risk of developing psychological problems themselves. Second, parents who have psychological problems and receive treatment are more prone to accept mental health treatment for their child [22]. In conclusion, relative to the general population, many multi-problem young adults often received mental health care during childhood and/or adolescence. However, if they were physically abused, there was a higher chance that they had not received mental health care. In other words, a subgroup with mental health care needs may not have received appropriate care. More adjusted individual screening on physical abuse and mental health care needs may be advised. It might also be beneficial to improve interventions that aim to support parents with mental health problems and to improve the parent–child relationship, as suggested by Chartier et al. [39] to decrease the occurrence of ACEs. Furthermore, to provide the needed care during childhood, it might also be beneficial to consider informal and community services. These forms of service use might be more accessible to families who perceive formal care services as stigmatizing [59, 61], which is likely the case in the disadvantaged families and neighbourhoods these multi-problem young adults grew up in. According to social stratification and social control theory, these disadvantaged communities lack social integration, have higher crime rates, and have less informal social control [35]. These circumstances may result in distrust and the alienation of residents from society, which could explain differences in norms and values, including deviant perceptions towards maltreatment, services such as police and youth care, mental health problems, and specialist mental health care use. The mismatch between the need and use of care services is also higher within these neighbourhoods than in advantaged neighbourhoods. To address this mismatch, Ellis and Dietz [96] introduced the Building Community Resilience (BCR) model [96]. This model aims to explore capacity issues of care organizations, reduce fragmented health care delivery, and facilitate integrated systems across partners. A community-based plan is worked out by care services to reduce and prevent trauma and toxic stress, improve physical and mental health, and build capacities that influence resilience. In the long term, our multi-problem young adult group might benefit more from such an approach instead of merely receiving formal mental health care during childhood. The last aim was to study the relation between ACEs, previous mental health care use, and current internalizing and externalizing problems of multi-problem young adults. This study confirmed previous results on the positive association, including a dose–response relationship, of ACEs and later mental health problems [6, 18, 39, 98]. More specifically, we showed that distinct types of ACEs related positively to mental health problems: multi-problem young adults who experienced emotional abuse were at higher risk for both internalizing and externalizing problems. This finding corresponds to the results of Hunt et al. [2], who found that emotional abuse increased internalizing and externalizing problems in children. However, another study showed a specific relation only with internalizing problems [19]. In contrast to what we expected, household dysfunction was not related to internalizing problems. Emotional neglect was found to be related to internalizing problems and was highly prevalent, which stresses the need for greater attention to emotional neglect in practice and future research. In addition, emotional abuse, physical abuse, and police contact of family members increased externalizing problems in multi-problem young adults. In alignment with previous study results, physical abuse and police contact of family members increases the risk of aggressive behaviour, inflicting pain and suffering on others, and antisocial and delinquent behaviour [1, 19, 99]. The different associations of emotional abuse and physical abuse with internalizing and externalizing problems relate to the conceptual framework that distinguishes between experiences of deprivation and threat. Emotional abuse could be considered deprivation, or the absence of expected environmental inputs and complexity, whereas physical abuse could be considered a threat, or the presence of experiences that represent a threat to one’s physical integrity. Deprivation and threat are considered to relate differently with neurodevelopment and functioning [100]. Thus, experiences of deprivation more often lead to the development of internalizing problems, and experiences of threat more often lead to the development of externalizing problems, based on the distinguished neurodevelopmental effects. When considering these results in light of the Stress Process Model [13, 14], primary stressors and moderating resources contribute to the association between ACEs and psychological functioning. In other words, it is possible that a primary stressor such as poverty leads to the secondary stressors of physical and emotional abuse, emotional neglect and police contacts within the family. Our results showed that in the long term, these secondary stressors are related to internalizing and externalizing problems. Therefore, to prevent these problems in young adulthood, it might be important to attend to primary stressors such as poverty during childhood instead of focusing only on ACEs. The present study had some limitations: first, the PCR data were collected regionally, therefore, we probably missed potential participants who moved to the Rotterdam Rijnmond region at a later age and had used mental health care. Second, we did not have information on care use other than that registered in the PCR. The register did not provide information on the care use of other services in the public sector such as school and social service care. However, it is highly likely that the multi-problem young adults in our sample had used other public health services or informal help. For example, we know that a large part (66%) of the sample was referred to the CPS as a consequence of severe family problems and/or juvenile delinquency [32]. Third, ACEs were related to problems in the family, e.g. psychological problems, but information on the registered mental health care use of family members was not available and would have enriched the study. Fourth, the PCR information was collected until 2010; hence, there was no information on the sample’s use of mental health services between 2010 and the time that they participated in this study. Registered mental health care use could, therefore, be underreported in this population. However, we did have information until adolescence (13 years) on all participants; 74.9% were 16 years or older. Prior mental health care use appeared not to be a strong predictor of current mental health problems, which may be partly due to the lack of the correspondence of mental health care use and the ACEs experienced by the young adults in our study. It is unknown to what extent the sample used other services that may be of more importance in reducing mental health problems. However, it is known that many young adults had contact with CPS and the juvenile justice system [54]. High-risk youth involved in the juvenile justice system, which is a comparable population to the one represented in our sample, had a lower likelihood of using professional mental health care but more often used informal services such as self-help, peer counselling groups, counselling from clergy or alternative healers [74]. In this respect, in addition to registering mental health care use, an extension to (informal) service use in future research is suggested. Fifth, self-report questionnaires were used to assess ACEs and current psychological problems. Specifically, abuse and neglect are sensitive topics. We increased the reliability of the questionnaire/procedure by asking the participants to fill out these questionnaires instead of assessing them verbally. A suggestion for future research is to explore the actual mental health problems in childhood and adolescence of multi-problem young adults; in addition to information on childhood experiences and mental health care use, the prevalence of child mental health problems would have extended this study. Sixth, we used a single ACE item approach that did not include important details, such as age of exposure, frequency, severity and duration [101], therefore, the findings must be interpreted with caution. However, this study provides more insight into the association between single ACEs relative to the other ACEs and outcomes. To address the cumulative burden of ACEs, we included analyses on the total number of ACEs. However, it should be noted that the sum of all ACEs does not actually capture the exact burden of ACEs because important details [101] were not measured in this study. More research on the type and timing of ACEs is recommended, as Schalinski et al. [101] showed that it improved the understanding of vulnerability to psychopathology. In addition, the different types of ACEs were all equally counted in the total ACE score, while the analyses with single ACE items showed that they are differentially associated with outcomes. Seventh, the ACE categories used in this study have a few exceptions relative to the general ACE study [4]; alcohol and drug abuse are examined separately instead of as one item of substance abuse, and police contact of family is examined more broadly. In conclusion, this study explored the pernicious long-term mental health consequences of ACEs in a high-risk multi-problem young adult group. Several ACEs were shown to be related to mental health care use in childhood and adolescence and to internalizing and externalizing problems in young adulthood. Cumulative ACE exposure associations with mental health problems confirmed these relations. A specific examination of tailored care for children who experienced emotional abuse, emotional neglect, physical abuse, and police contact with family members is needed. Emotional abuse and emotional neglect are often chronic and difficult to detect [102], and physically abused children may have an unmet service need. Therefore, special attention and screening on these adverse events is recommended. In addition, screening and intervening in psychological problem development in children experiencing ACEs may be beneficial to prevent later mental health problems. Lastly, prevention, decreases in poverty, and a community approach are recommended to increase the effects of (informal) care in disadvantaged neighbourhoods.

## Abbreviations

ACE: Adverse childhood experience
ASR: Adult Self-Report
AUC: Area under the curve
BCR: Building Community Resilience
CTQ-SF: Childhood Trauma Questionnaire-Short Form
Df: Degrees of freedom
DNK: New Opportunities (Dutch: *De Nieuwe Kans*)
HMO: Health maintenance organization
PCR: Psychiatric case register
SD: Standard deviation
SPSS: Statistical Packages for the Social Sciences
SSM-D: Self-Sufficiency Matrix—Dutch version
WHO: World Health Organization

## Footnotes

1 The response rate was 56%.

2 The response rate was 62%.

3 Registration number: 2013.422—NL46906.029.13.
